# Low‐Dose naltrexone restored TRPM3 ion channel function in natural killer cells from long COVID patients

**DOI:** 10.3389/fmolb.2025.1582967

**Published:** 2025-05-19

**Authors:** Etianne Martini Sasso, Natalie Eaton-Fitch, Peter Smith, Katsuhiko Muraki, Sonya Marshall-Gradisnik

**Affiliations:** ^1^ The National Centre for Neuroimmunology and Emerging Diseases, Griffith University, Gold Coast, QLD, Australia; ^2^ Consortium Health International for Myalgic Encephalomyelitis, National Centre for Neuroimmunology and Emerging Diseases, Griffith University, Gold Coast, QLD, Australia; ^3^ School of Pharmacy and Medical Sciences, Griffith University, Gold Coast, QLD, Australia; ^4^ Queensland Allergy Services, Gold Coast, Australia; ^5^ Laboratory of Cellular Pharmacology, School of Pharmacy, Aichi-Gakuin University, Nagoya, Japan

**Keywords:** calcium, TRP ion channels, TRPM3, long COVID, low-dose naltrexone, Transient Receptor Potential Melastatin 3

## Abstract

**Introduction:**

Long COVID is a multisystemic condition that includes neurocognitive, immunological, gastrointestinal, and cardiovascular manifestations, independent of the severity or duration of the acute SARS-CoV-2 infection. Dysfunctional Transient Receptor Potential Melastatin 3 (TRPM3) ion channels are associated with the pathophysiology of long COVID due to reduced calcium (Ca^2+^) influx, negatively impacting cellular processes in diverse systems. Accumulating evidence suggests the potential therapeutic benefits of low-dose naltrexone (LDN) for people suffering from long COVID. Our study aimed to investigate the efficacy of LDN in restoring TRPM3 ion channel function in natural killer (NK) cells from long COVID patients.

**Methods:**

NK cells were isolated from nine individuals with long COVID, nine healthy controls, and nine individuals with long COVID who were administered LDN (3–4.5 mg/day). Electrophysiological experiments were conducted to assess TRPM3 ion channel functions modulated by pregnenolone sulfate (PregS) and ononetin.

**Results:**

The findings from this current research are the first to demonstrate that long COVID patients treated with LDN have restored TRPM3 ion channel function and validate previous reports of TRPM3 ion channel dysfunction in NK cells from individuals with long COVID not on treatment. There was no significant difference in TRPM3 currents between long COVID patients treated with LDN and healthy controls (HC), in either PregS-induced current amplitude (p > 0.9999) or resistance to ononetin (p > 0.9999).

**Discussion:**

Overall, our findings support LDN as a potentially beneficial treatment for long COVID patients by restoring TRPM3 ion channel function and reestablishing adequate Ca^2+^ influx necessary for homeostatic cellular processes.

## 1 Introduction

Long COVID is an emerging multisystemic disorder that occurs after acute infection with respiratory syndrome coronavirus 2 (SARS-CoV-2). It is estimated that at least 10% of individuals who contract COVID-19 will develop long COVID (The Lancet Infectious Diseases, 2023; The Lancet, 2023; Davis et al., 2023). Due to the debilitating and persistent symptoms that affect most organ systems, long COVID is associated with poor quality of life (QoL), and individuals experience a significant impact on their social and work function (Komaroff and Lipkin, 2023; Davis et al., 2021; Weigel et al., 2024). The most common symptoms reported in long COVID include fatigue, cognitive problems, neuropsychiatric symptoms, sleep disturbances, headaches, gastrointestinal disturbances, post-exertional malaise, pain, and cardiovascular and pulmonary abnormalities (Davis et al., 2021; Lopez-Leon et al., 2021; Carfi et al., 2020; Mantovani et al., 2021; World Health Organization, 2021a). Although some factors increase the risk of developing long COVID, such as virus variants, hospitalization during SARS-CoV-2 infection, and reinfection, long COVID occurs in all age groups, genders, and ethnicities, regardless of the initial infection severity (Al-Aly et al., 2024). Furthermore, follow-up studies have reported that long COVID symptomatology persists for over 2 years (Kim et al., 2023; Huang et al., 2022; Mateu et al., 2023).

The immunopathology of SARS-CoV-2 infection is characterized by the dysregulation of both innate and adaptive immune responses (Zheng et al., 2020; Wang et al., 2020; van Eeden et al., 2020; Mustafa et al., 2025), with a significant increase in cytokine levels observed in the most severe COVID-19 cases—a phenomenon known as the “cytokine storm” (Varchetta et al., 2021; Queiroz et al., 2022; Low et al., 2023). The impairment of the immune system is also suggested by the lymphopenia observed in COVID-19 patients during infection, as well as by the association of a decline in natural killer (NK) cell counts and distinct NK cell immunotypes with COVID-19 severity (Zheng et al., 2020; Wang et al., 2020; Varchetta et al., 2021; Maucourant et al., 2020). Similarly, studies have demonstrated that immune dysregulation is a key feature in the pathophysiology of long COVID, with underlying mechanisms varying according to the long COVID phenotype, the severity of the acute infection, and the tissues or organs involved (Gomes et al., 2023; Sanchez-Menendez et al., 2024; Yin et al., 2024; Mohandas et al., 2023; Altmann et al., 2023). A recent publication identified transcriptome alteration in long COVID patients, with fifteen upregulated and fourteen downregulated genes, suggesting abnormalities in the expression of genes involved in antigen presentation, cytokine signaling, and immune cell activation (Eaton-Fitch et al., 2024). Data from a plasma protein investigation indicated that NK cells in individuals with long COVID exhibit a shift from an activated to a resting phenotype, based on reduced expression of CC-chemokine receptor 7 markers (Iosef et al., 2023).

While the impact of COVID-19 has been alleviated through vaccine development and increased clinical experience in managing acute infection, the pathomechanism and potential pharmacotherapeutic interventions for long COVID are yet to be determined (The Lancet, 2023; Davis et al., 2023; Boccellino, 2023). Given that the pathomechanism of long COVID remains inconclusive, numerous studies have consistently reported overlaps with myalgic encephalomyelitis/chronic fatigue syndrome (ME/CFS) (Komaroff and Lipkin, 2023; Mantovani et al., 2021; Gonzalez-Hermosillo et al., 2021; Wong and Weitzer, 2021; Sukocheva et al., 2021; Malkova et al., 2021; Yong and Liu, 2021). ME/CFS is another debilitating, multifactorial, acquired illness that affects the neurological, endocrine, immunological, and gastrointestinal systems. It is also accompanied by impaired cellular energy metabolism and ion transport (Rasa et al., 2018). Evidence demonstrates that NK cells from individuals diagnosed with ME/CFS and long COVID exhibit similar dysfunction in the Transient Receptor Potential Melastatin 3 (TRPM3) ion channel, which may help explain the array of symptoms experienced by these patients groups (Sasso et al., 2022).

TRPM3 ion channel is highly permeable to calcium (Ca^2+^) and shares typical features with other TRP ion channels, including six transmembrane domains, a pore domain, and a TRP domain located at the C-terminal (Grimm et al., 2003; Thiel et al., 2017). Notably, the TRPM3 ion channel is expressed in many cell types of the human organism, with high tissue expression in the central nervous system, kidneys, liver, pancreatic beta islets, cardiovascular system, skeletal muscle, genitourinary tract, testes, ovaries, spinal cord, and immune cells (Montell et al., 2002; Nguyen et al., 2016; Nilius and Owsianik, 2011; Li, 2017; Thiel et al., 2013). Moreover, TRPM3 affects Ca^2+^ signaling, contributing to biological processes, intracellular pathways, and cellular homeostasis (Schwarz et al., 2013; Clapham, 2007). In contrast, TRPM3 ion channel dysfunction is associated with impaired cell function due to disturbances in intracellular Ca^2+^ signals (Cabanas et al., 2018; Eaton-Fitch et al., 2022; Cabanas et al., 2019a). Lohn and Wirth published a recent review that mainly focused on TRPM3 involvement in ME/CFS, while also exploring the role of TRPM3 dysfunction in long COVID and the potential benefits of low‐dose naltrexone (LDN) (Lohn and Wirth, 2024).

Naltrexone hydrochloride (NTX) is a competitive mu (μ)-opioid receptor antagonist, primarily prescribed for the treatment of alcohol or opioid dependence at daily doses of 50–100 mg (Bolton et al., 2020; Bolton et al., 2019; Polo et al., 2019; Toljan and Vrooman, 2018; Cabanas et al., 2021; Sharafaddinzadeh et al., 2010; Cree et al., 2010; Younger et al., 2009; Liu et al., 2016; Smith et al., 2011). Opioid receptors are members of the guanine nucleotide-binding protein-coupled receptor (GPCR) superfamily and are widely distributed in human tissues, including the central nervous system, gastrointestinal tract, and immune cells (Herman et al., 2024; Ninkovic and Roy, 2013; Sobczak et al., 2014; Peng et al., 2012). Earlier investigations revealed promising outcomes regarding the potential of NTX to restore TRPM3 function, based on *in vitro* experiments using NK cells from both ME/CFS and long COVID patients (Cabanas et al., 2019b; Sasso et al., 2024).

A dose of NTX greater than 50 mg/day inhibits the effects of endorphins that sustain addiction, while a low dose (≤5 mg/day, also referred to as LDN) exerts anti-inflammatory and immunomodulatory effects, in addition to modulating pain sensitivity—mechanisms that substantiate the hypothesis that LDN alleviates symptoms in a range of chronic conditions (Polo et al., 2019; Choubey et al., 2022; Patten et al., 2018). Off-label use of LDN has been reported to be beneficial for patients with Crohn’s disease, fibromyalgia, multiple sclerosis, complex-regional pain syndrome, Hailey–Hailey disease, ME/CFS, and cancer (Bolton et al., 2020; Polo et al., 2019; Toljan and Vrooman, 2018; Liu et al., 2016; Patten et al., 2018). Overall, LDN has been associated with symptom relief frequently seen in long COVID and a low incidence of adverse effects (Dietz and Brondstater, 2024; O'Kelly et al., 2022).

Based on estimates that 10% of individuals infected with SARS-CoV-2 develop long COVID (Davis et al., 2023) and that the World Health Organization (WHO) reported over 777 million COVID-19 cases as of March 2025 (World Health Organization, 2021b), the global prevalence of long COVID exceeded 77 million people. However, the prevalence is likely underestimated, as not all cases are reported to the WHO, due to the reduction in testing rates and the high frequency of asymptomatic and mild cases after the advent of vaccination. Therefore, there is an urgent need to identify potential treatments that mitigate long COVID impacts on society and healthcare systems. Given TRPM3 ion channel has been identified as a biomarker in the pathophysiology of long COVID (Sasso et al., 2022; Sasso et al., 2024), in addition to the efficacy of *in vitro* treatment with NTX to restore TRPM3 ion channels in cells from long COVID individuals (Sasso et al., 2024) and the potential of LDN to alleviate symptoms of this condition (O'Kelly et al., 2022; Hurt et al., 2024; Bonilla et al., 2023), this study aimed to assess the TRPM3 ion channel function in NK cells from long COVID patients undergoing LDN treatment to evaluate the potential effectiveness of this drug in restoring TRPM3 ion channel function.

## 2 Materials and methods

### 2.1 Participant characteristics

In this study, 27 volunteers were screened and recruited through the National Centre for Neuroimmunology and Emerging Diseases (NCNED) patient database from 6 March 2023 to 17 September 2024. Eligible participants were 18–65 years old and had no previous diagnosis of diabetes, autoimmune diseases, thyroid disorders, primary psychiatric illnesses, or cardiovascular disease. This investigation included three participant groups: 1) individuals with long COVID (N = 9); 2) healthy controls (HC; N = 9); 3) individuals with long COVID receiving LDN (N = 9). The HC group consists of individuals in good health, with no symptoms of fatigue or evidence of illness, and no history of suspected or confirmed SARS-CoV-2 infection.

Participants in both the long COVID and long COVID taking LDN groups had previously been diagnosed with long COVID and met the WHO clinical criteria by the Delphi Consensus (World Health Organization, 2021a). To be included in the LDN group, participants must be in treatment with a daily dose of 3 to 5 mg of NTX for at least 4 weeks (prescribed by their physicians). Individuals were excluded if they reported alcohol abuse, smoking, pregnancy, breastfeeding, obesity (body mass index (BMI) ≥ 30), or the use of opioids, medications, or supplements that affect TRPM3 activity or Ca^2+^ signaling. Participants had the option to temporarily discontinue interfering medications or supplements, in accordance with their half-life and after authorized by their physician. The Griffith University Human Research Ethics Committee approved this study (GU HREC 2022/666), and all participants provided written informed consent.

### 2.2 Participant symptoms and disability

Initially, participants completed an online questionnaire to provide sociodemographic information, medical history, medication use, and patient-reported outcome measures, including the 36-item Short-Form Health Survey (SF-36) (Ware, 2000) and the WHO Disability Assessment Schedule (WHODAS) (Üstün et al., 2022).

The SF-36 was evaluated using internationally validated measures, which combined items within the same domain into eight scale scores: physical functioning, role limitations due to physical health problems, pain, general health, vitality, social functioning, role limitations due to personal or emotional problems, and emotional wellbeing. SF-36 domains were scored on a scale of 0% to 100%, with higher scores indicating higher QoL (Ware, 2000).

WHODAS, which is subdivided into seven domains assessed communication and understanding, mobility, self-care, interpersonal relationships, life activities, participation in society, and work or school participation. First, WHODAS items were scored on a five-point scale (none = 0, mild = 1, moderate = 2, severe = 3, and extreme or cannot do = 4). Subsequently, scores were converted from 0% to 100%, with lower scores indicating less disability and 100% corresponding to full disability (Üstün et al., 2022).

### 2.3 Peripheral blood mononuclear cell and NK cell isolation

In this research, we used NK cells based on the consistent findings of immune disruption in long COVID patients, prior validation of the NK cell model in ME/CFS research (Cabanas et al., 2018; Eaton-Fitch et al., 2022; Cabanas et al., 2019a; Eaton-Fitch et al., 2021; Eaton-Fitch et al., 2019) and evidence indicating impaired endogenous TRPM3 ion channel function in NK cells from long COVID patients (Sasso et al., 2022; Sasso et al., 2024). The NK model can be further justified using a recent meta-analysis by Baraniuk et al. (2024). Hence, to study the effectiveness of LDN in restoring TRPM3 ion channel function, we selected a validated model to continue our research. Furthermore, NK cells offer the advantage of being acquired *via* venipuncture of blood samples, causing low discomfort and risks for participants, in contrast with more invasive procedures involved in biopsy collections of muscle or other organ/tissue cells (Eaton-Fitch et al., 2019; Brenu et al., 2011; Bansal et al., 2012; Brenu et al., 2014).

Each participant provided up to 84 mL of whole blood drawn through *via* venipuncture in ethylenediaminetetraacetic acid (EDTA) tubes. A portion of the blood sample was sent to the pathology laboratory for a full blood count (FBC). Peripheral blood mononuclear cells (PBMC) were isolated from the remaining sample by centrifugation over a density gradient medium (Ficoll–Paque Premium, GE Healthcare, Uppsala, Sweden). PBMC total cell count, lived cell count, and viability were obtained using trypan blue dye (Invitrogen, Carlsbad, CA, United States) and an automatic cell counter (TC20 Automated cell counter, Bio-Rad, Laboratories, Hercules, CA). Immediately after PBMC isolation, NK cells were isolated using immunomagnetic selection and EasySep Negative Human NK Cell Isolation Kit (Stem Cell Technologies, Vancouver, BC, Canada). Flow cytometry was performed to assess NK cell purification. The NK cell population was then detected by phenotypic surface expression of CD3^–^CD56^+^ using the BD LSRFortessa™ X-20 flow cytometer (BD Biosciences, San Diego, CA, United States). Isolated cells were incubated for 20 min with CD56 APC (0.25 g/20 μL) and CD3 PE Cy7 (0.25 g/5 μL) monoclonal antibodies (Becton Dickinson (BD) Bioscience, San Jose, CA, United States) and 10,000 events were acquired after cells were washed and resuspended in stain buffer (BD Bioscience, New Jersey, United States). Supplementary Figure S1 shows the gating strategy and purity results from each group.

### 2.4 Electrophysiological experiments

Whole-cell patch-clamp experiments were performed on freshly isolated NK cells from all participants to investigate endogenous TRPM3 ion channel function in people with long COVID treated with LDN, compared to HC and individuals with long COVID not receiving LDN treatment. The intracellular pipette solution in this study consisted of 30 mM CsCl, 2 mM MgCl_2_, 110 mM L-Aspartic acid, 1 mM EGTA, 10 mM HEPES, 4 mM ATP disodium hydrate, 0.1 mM GTP sodium salt hydrate (pH = 7.2, adjusted with CsOH; osmolality = 290 mOsm/L, adjusted with D-mannitol), filtered with a 0.22-μm membrane filter and stored at −20 °C. L-aspartic acid was added to this solution to reduce the risk of chloride current involvement in TRPM3 assessment. Its inclusion allowed to distinguish the reversal potential of Cl^-^ currents at −37.24 mV from that of cation currents (approximately 0 mV), based on the Nernst equation. The extracellular solution was freshly prepared with 130 mM NaCl, 10 mM CsCl, 1 mM MgCl_2_, 1.5 mM CaCl_2_ 2H_2_O, 10 mM HEPES, (pH = 7.4, adjusted with NaOH; osmolality = 300 mOsm/L, adjusted with D-glucose).

In this study, glass pipettes were pulled using borosilicate glass capillaries (Harvard Apparatus, Holliston, MA, United States, GC150F-15, outside diameter = 1.5 mm, inside diameter = 0.86 mm) and polished immediately before use. Pipette resistance was 8–12 MΩ, and we used a CV203BU head-stage (Molecular Devices, Sunnyvale, CA, United States) connected to a 3-way coarse manipulator and a micro-manipulator (Narishige, Tokyo, Japan). An Axopatch 200B amplifier and pClamp 10.7 software (Molecular Devices, Sunnyvale, CA, United States) were used to amplify and record electrical signals, with data filtered at 5 kHz and sampled digitally at 10 kHz *via* a Digidata 1440A analog-to-digital converter (Molecular Devices, Sunnyvale, CA, United States). The voltage-ramp protocol consisted of a step from a holding potential of +10 mV to −90 mV, followed by a 0.1 s ramp to +110 mV, before returning to +10 mV (repeated every 10 seconds). The liquid junction potential between the pipette and bath solutions (−10 mV) was corrected, and no leak current component was subtracted.

The pharmacological TRPM3 modulation protocol consisted of 50 seconds of the extracellular solution to set a baseline, followed by application of pregnenolone sulfate (PregS) at 100 μm in the same solution and subsequently by 150 seconds of ononetin at 10 μm in the presence of PregS at 100 μm. The protocol was finalized with a wash-out of 100 seconds with only extracellular solution. Pharmacomodulators (PregS and ononetin) were purchased from Tocris Bioscience, resuspended, and stored according to the manufacturer’s instructions. All patch-clamp experiments were performed at room temperature (22–24°C).

### 2.5 Statistical analysis

Statistical Package for the Social Sciences (SPSS) version 27 (IBM Corp, Armonk, NY, United States) was used for analyzing the questionnaire data, while flow cytometry results analyzed using GraphPad Prism version 9 (GraphPad Software Inc., La Jolla, CA, United States). All electrophysiological experiments were individually analyzed by a blind researcher and reviewed by a second blind researcher to ensure quality control. Any recordings with unstable currents or Cl^−^ contamination were excluded from the statistical analysis. Moreover, pCLAMP 10.7 software (Molecular Devices, Sunnyvale, CA, United States), Origin 2021 (OriginLab Corporation, Northampton, MA, United States), and GraphPad Prism version 9 were used for analyzing patch-clamp data. We conducted the Shapiro–Wilk normality test to identify the distribution of data and the ROUT method to detect and remove outliers. Statistical comparisons between groups for noncategorical variables (agonist and antagonist amplitudes) were conducted using the independent nonparametric Kruskal–Wallis test (Dunn’s multiple comparisons). Categorical variables (sensitivity to ononetin) were analyzed using Fisher’s exact test (Bonferroni method). Power calculations using G*Power were included to support the sample size of previous studies (Cabanas et al., 2018; Cabanas et al., 2019a; Cabanas et al., 2019b). Based on the mean and SD from these studies, a sample size greater that n = 5 per participant per group was determined to provide sufficient statistical power using the following parameters: (i) effect size of 0.5, (ii) type 1 error of 5% (α = 0.05), and (iii) power of 80%. A p-value of < 0.05 was considered statistically significant, and data were presented as mean ± standard error of the mean (SEM), unless otherwise stated.

## 3 Results

### 3.1 Participant demographics, symptoms, and blood parameters

A total of 27 participants were included in this study, subdivided into three groups: N = 9 HC, N = 9 long COVID, and N = 9 long COVID treated with LDN. Among HC participants, the mean age and standard derivation (SD) were 42.22 ± 10.51 years and seven were women (77.8%). In the long COVID group, the mean age was 43.00 ± 12.48 years, and four were women (44.4%), while for long COVID participants treated with LDN, the mean age was 57.56 ± 7.764 years, and seven were women (77.8%). A statistically significant difference (p = 0.006) in age was observed among groups, primarily due to the older average age in the LDN group compared to other groups. In contrast, no statistical difference was identified regarding gender, BMI, employment status, education level, and FBC parameters (except hematocrit), and these data are summarized by each group in Table 1.

**TABLE 1:** Participant demographics and FBC parameters.

		LC	HC	LC receiving LDN	P-value
Age (years)		43.00 ± 12.48	42.22 ± 10.51	57.56 ± 7.764	**0.006**
Gender N (%)	Female Male	4 (44.4%) 5 (55.6%)	7 (77.8%) 2 (22.2%)	7 (77.8%) 2 (22.2%)	0.236
BMI (kg/m^2^)		23.91 ± 2.966	24.26 ± 3.122	26.13 ± 2.230	0.216
Employment Status	Full time	5 (55.6%)	6 (66.7%)	6 (66.7%)	0.518
	Part time	3 (33.3%)	2 (22.2%)	1 (11.1%)	
	Casual	0 (0.0%)	1 (11.1%)	0 (0.0%)	
	Unemployed	1 (11.1%)	0 (0.0%)	2 (22.2%)	
	Illness/disability	0 (0.0%)	0 (0.0%)	0 (0.0%)	
Education	Primary education	0 (0.0%)	0 (0.0%)	0 (0.0%)	0.897
	High school	0 (0.0%)	1 (11.1%)	0 (0.0%)	
	Undergraduate	4 (44.4%)	4 (44.4%)	7 (77.8%)	
	Postgraduate/doctoral	4 (44.4%)	3 (33.3%)	1 (11.1%)	
	Professional training	1 (11.1%)	1 (11.1%)	1 (11.1%)	
Full Blood Count	White cell count (4.0–11.0 × 10^9^/l)	5.69 ± 1.73	5.93 ± 0.91	6.31 ± 1.97	0.389
	Lymphocytes (1.0–4.0 × 10^9^/L)	1.82 ± 0.70	1.73 ± 0.30	1.76 ± 0.49	0.893
	Neutrophils (2.0–8.0 × 10^9^/L)	3.16 ± 0.91	3.56 ± 0.74	3.88 ± 1.99	0.565
	Monocytes (0.1–1.0 × 10^9^/L)	0.40 ± 0.10	0.46 ± 0.11	0.45 ± 0.08	0.600
	Eosinophils (<0.6 × 10^9^/L)	0.25 ± 0.15	0.13 ± 0.05	0.16 ± 0.14	0.119
	Basophils (<0.2 × 10^9^/L)	0.04 ± 0.03	0.05 ± 0.02	0.06 ± 0.03	0.405
	Platelets (140–400 × 10^9^/L)	264.89 ± 37.58	247.44 ± 72.70	267.89 ± 54.98	0.819
	Red cell count (3.8–5.2 × 10^12^/L)	4.88 ± 0.48	4.48 ± 0.44	4.43 ± 0.26	0.112
	Hematocrit (0.33–0.47)	0.43 ± 0.03	0.41 ± 0.04	0.40 ± 0.02	**0.042** ^**a**^
	Hemoglobin (115–160 g/L)	143.00 ± 13.05	135.22 ± 11.85	133.00 ± 7.48	0.226

Data presented as mean ± SD or N (%) and determined by Kruskal–Wallis test. Values of p < 0.05 are bolded. Signal a indicates only a significant difference between long COVID and long COVID receiving LDN groups. Reference ranges for full blood count parameters have been included in the table. Abbreviations: BMI, body mass index; FBC, full blood count; HC, healthy controls; LC, long COVID; LDN, low-dose naltrexone.

Participants’ QoL and disability were assessed using the SF-36 and WHODAS questionnaires. Table 2 presents the details of eight domains from SF-36 on a scale from 0% to 100%, whereby scores are directly proportional to QoL and seven domains from WHODAS, whereby 100% indicates total disability. First, when comparing three groups, significant differences were observed in six out of eight SF-36 domains (physical functioning, physical role, pain, general health, social functioning, and vitality, as well as all scores from WHODAS (communication and understanding, mobility, self-care, interpersonal relationships, life activities, work activities, and participation in society). Second, when comparing the long COVID group with the long COVID group receiving LDN, a significant difference was observed only in the mobility domain. Finally, when comparing HC and the long COVID group receiving LDN, no significant differences were observed regarding general health, emotional role, emotional well-being, vitality, self-care, interpersonal relationships, and work activities.

**TABLE 2:** Participant quality of life and disability scores.

	LC	HC	LC receiving LDN	P-value among 3 groups	P-value LC and LC receiving LDN	P-value HC and LC receiving LDN
SF-36 (%)						
Physical functioning	48.89 ± 29.24	100 ± 0.00	62.14 ± 21.38	**<0.001**	0.486	**<0.001**
Physical role	26.39 ± 26.66	100 ± 0.00	34.82 ± 29.94	**<0.001**	0.455	**<0.001**
Pain	52.78 ± 26.82	97.50 ± 4.63	67.50 ± 23.63	**0.001**	0.285	**0.002**
General health	32.41 ± 16.24	76.56 ± 10.67	50.00 ± 24.88	**0.002**	0.164	0.063
Social functioning	27.78 ± 27.79	93.75 ± 17.68	48.21 ± 30.13	**0.001**	0.163	**0.006**
Emotional role	74.07 ± 29.30	94.79 ± 14.73	70.24 ± 30.37	0.115	0.785	0.073
Emotional well-being	53.33 ± 16.77	72.50 ± 22.52	60.71 ± 20.09	0.156	0.365	0.199
Vitality	22.22 ± 19.79	66.41 ± 18.88	39.28 ± 29.25	**0.007**	0.241	0.062
WHODAS (%)						
Communication & understanding	33.33 ± 11.02	5.73 ± 13.16	26.78 ± 16.98	**0.007**	0.489	**0.020**
Mobility	35.55 ± 22.42	0.00 ± 0.00	12.14 ± 11.49	**0.001**	**0.029**	**0.006**
Self-care	16.67 ± 24.61	0.00 ± 0.00	1.78 ± 3.05	**0.013**	0.084	0.117
Interpersonal relationships	33.33 ± 17.68	5.47 ± 10.26	16.07 ± 17.25	**0.004**	0.053	0.066
Life activities	51.39 ± 26.29	0.00 ± 0.00	33.93 ± 18.35	**<0.001**	0.134	**<0.001**
Work activities	44.44 ± 34.01	9.37 ± 21.91	39.28 ± 31.60	**0.044**	0.957	0.068
Participation in society	42.71 ± 22.75	3.91 ± 9.85	41.96 ± 21.49	**0.001**	0.874	**0.003**

Data presented as mean ± SD and determined by Kruskal–Wallis test. Missing data from N = 1 HC and N = 2 long COVID receiving LDN. Values of p < 0.05 are bolded. Abbreviations: HC, healthy controls; LC, long COVID; LDN, low-dose naltrexone; SF-36, 36-item Short-Form Health Survey; WHODAS, World Health Organization disability assessment schedule.

In the long COVID on LDN treatment group, the median duration of treatment was 7.2 months, with an LDN average dose of 4 mg/day (N = 3 on 3 mg/day and N = 6 on 4.5 mg/day).

### 3.2 Electrophysiological experiments

Using the gold standard technique of whole-cell patch-clamp, TRPM3 ion channel function was stimulated with 100 μM PregS, while its activation was blocked using a consecutive application of 10 μM ononetin in the presence of PregS (Cabanas et al., 2018; Naylor et al., 2010; Majeed et al., 2010; Persoons et al., 2021; Wagner et al., 2008; Vanneste et al., 2021; Alonso-Carbajo et al., 2019; Straub et al., 2013). In the electrophysiological experiments, we included nine participants in each group and analyzed recordings from 61, 65, and 63 independent cells for PregS effects from long COVID, HC, and long COVID receiving LDN groups, respectively. In addition, to assess ononetin effects in the presence of PregS, 52 independent recordings from NK cells in the long COVID group, 53 in NK cells from HC, and 53 recordings from NK cells in the long COVID group receiving LDN.

**FIGURE 1:**
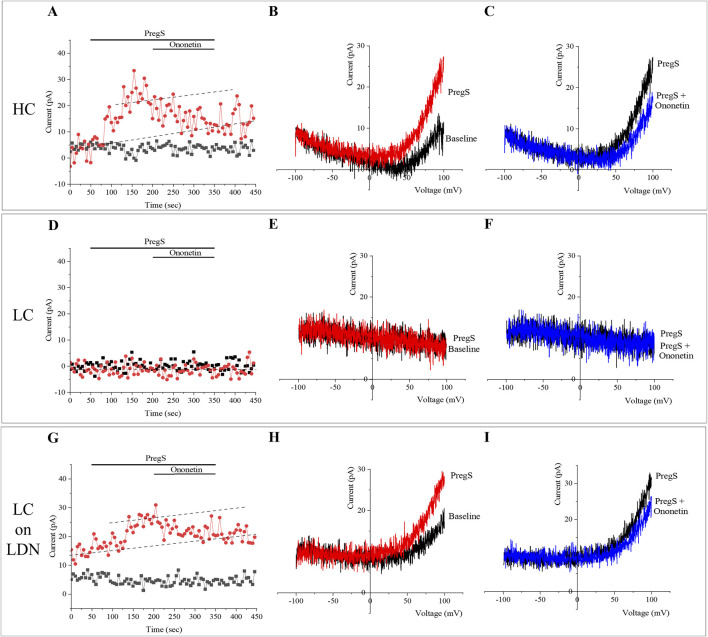
TRPM3 ion channel function in NK cells modulated with PregS and ononetin. **(A–C)** Current obtained on an NK cell from an HC participant. **(A)** A representative time-series of current amplitude at +100 mV and −100 mV. **(B)** I–V curve before (baseline) and after TRPM3 stimulation through PregS application. **(C)** I–V curve on PregS stimulation and after TRPM3 inhibition with ononetin in the presence of PregS. **(D–F)** Current obtained on an NK cell from a long COVID participant. **(D)** A representative time-series of current amplitude at +100 mV and −100 mV. **(E)** I–V curve before (baseline) and after TRPM3 stimulation through PregS application. **(F)** I–V curve on PregS stimulation and after TRPM3 inhibition with ononetin in the presence of PregS. **(G–I)** - Current obtained on an NK cell from a long COVID participant on treatment with LDN. **(G)** A representative time-series of current amplitude at +100 mV and −100 mV. **(H)** I–V curve before (baseline) and after TRPM3 stimulation through PregS application. **(I)** I–V curve on PregS stimulation and after TRPM3 inhibition with ononetin in the presence of PregS. Dash lines in **(A,D,G)** illustrate each trend of the baseline and PregS effects. Abbreviations: HC, healthy control; LC, long COVID; LDN, low-dose naltrexone; NK, natural killer; PregS, pregnenolone sulfate.

**FIGURE 2:**
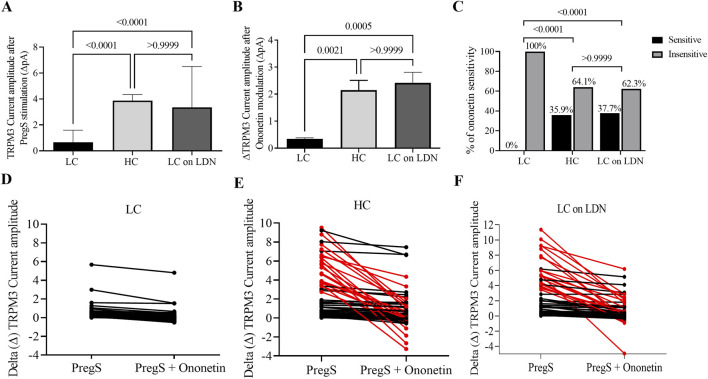
TRPM3 ion channel function comparison among long COVID, HC and long COVID taking LDN groups. **(A)** Bar graph showing TRPM3 current amplitude at +100 mV after TRPM3 stimulation through PregS application in long COVID (N = 9; n = 61), HC (N = 9; n = 65) and long COVID taking LDN (N = 9; n = 63). **(B)** Bar graphs illustrating TRPM3 inhibition with ononetin in the presence of PregS in long COVID (N = 9; n = 52), HC (N = 9; n = 53) and long COVID taking LDN (N = 9; n = 53). **(C)** Bar graphs representing the percentage of NK cells sensitive and insensitive to ononetin inhibition in the presence of PregS. **(D–F)** Scatter plots showing change of each current recording in NK cells from long COVID, HC and long COVID taking LDN groups, respectively; first point was measured at the end of TRPM3 stimulation through PregS application, and the second point shows the effect in current amplitude after TRPM3 inhibition with ononetin in the presence of PregS. Each red line represented a cell sensitive to ononetin as a reduction in amplitude was recorded. N = participants and n = number of records. Data presented as mean ± SEM and determined by Kruskal–Wallis (Dunn’s multiple comparisons, **(A,B)** in amplitude analysis and Fisher’s exact test (Bonferroni method, C) regarding ononetin response. Abbreviations: HC, healthy control; LC, long COVID; LDN, low-dose naltrexone; N, number of participants; n, number of records analyzed; NK, natural killer; PregS, pregnenolone sulfate.

In normal cellular conditions, the TRPM3 agonist PregS activates the TRPM3 ion channels in the plasma membrane, leading to an increase in Ca^2+^ influx into the cells. For instance, in NK cells from the HC group, we observed PregS-induced outwardly rectifying current under voltage-clamp conditions (Figure 1A), also identifying usual TRPM3 current–voltage relationship (I–V) curve (Figure 1B) in most cells. In contrast, the same PregS concentration only activated TRPM3 currents in a few NK cells in the long COVID group, as illustrated in Figures 1D,E. Consistent with previous studies, PregS-induced TRPM3 currents were significantly reduced in NK cells from the long COVID group compared to HC (p < 0.0001). Interestingly, this investigation also identified statistical differences comparing both long COVID groups (p < 0.0001), with an increase in PregS amplitudes in the long COVID group receiving LDN treatment. These values did not differ significantly between the long COVID receiving LDN and the HC group (p > 0.9999), which demonstrated TRPM3 ion channel function was reestablished by treatment with LDN. Figure 2A shows statistical results for PregS-induced TRPM3 currents.

In line with prior publications, ononetin effectively inhibited PregS-induced currents in 64.1% of NK cells from the HC group but had no effect on NK cells from the long COVID group (0% NK cells were sensitive to ononetin). Interestingly, the long COVID group receiving LDN had a similar rate (62.3%) of PregS-induced TRPM3 currents suppressed by ononetin as seen in the HC group. Figure 1 presents representative I-V curves during PregS application and after inhibition with ononetin in the HC group (Figure 1C), the long COVID group (Figure 1F), and the long COVID group receiving LDN treatment (Figure 1I). As reported in earlier studies, we confirmed a reduction in ononetin amplitude (p = 0.0021) and the number of cells sensitive to ononetin (p < 0.0001) when compared to the HC and long COVID group. In contrast, NK cells from the long COVID group receiving LDN had a significant elevation in amplitude (p = 0.0005) and sensitivity (p < 0.0001) to ononetin compared with the long COVID group. Moreover, no statistical difference was observed between the long COVID group receiving LDN and the HC group in response to ononetin (p > 0.9999), which confirms the effectiveness of LDN in restoring TRPM3 ion channel activity in NK cells from long COVID patients. Figures 2B–F summarize the results of ononetin application and statistical comparisons among groups.

## 4 Discussion

In this study, we performed whole-cell patch-clamp experiments to investigate the effects of LDN on TRPM3 ion channel activity in NK cells freshly isolated from long COVID patients receiving LDN treatment. Our results provide the first evidence of LDN benefits in restoring TRPM3 ion channel function in NK cells from long COVID patients. As demonstrated, the application of TRPM3 agonist (PregS) stimulated typical TRPM3 current outward rectification, while the antagonist (ononetin) suppressed PregS-induced currents in NK cells from long COVID patients treated with LDN, mimicking results reported in HC individuals. Importantly, no statistical differences were found comparing PregS-induced TRPM3 currents from long COVID receiving LDN with HC in neither PregS-induced TRPM3 currents amplitude (p > 0.9999) or the resistance to ononetin (p > 0.9999), which confirms TRPM3 was reestablished in those long COVID patients taking LDN treatment.

Restoration of TRPM3 function in NK cells suggests the reestablishment of proper Ca^2+^ signaling pathways, which directly impact immune regulation and cellular function in long COVID patients (Schwarz et al., 2013; Clapham, 2007). Consequently, TRPM3 restoration in NK cells, and potentially other immune cells, helps address the immune dysregulation associated with long COVID (Cabanas et al., 2018; Eaton-Fitch et al., 2022; Cabanas et al., 2019a). Moreover, it is essential to highlight that TRPM3 is expressed in a wide variety of cell types, contributing to diverse physiological functions associated with symptoms in individuals with long COVID (Montell et al., 2002; Nguyen et al., 2016; Nilius and Owsianik, 2011; Li, 2017; Thiel et al., 2013). While the NK cell model offers the advantage of being obtained from blood samples and minimizes risk and discomfort for participants, future studies should investigate TRPM3 function in other cell types and tissues. This will help identify whether Ca^2+^ influx is similarly restored during LDN treatment and whether such restoration contributes to the recovery of other systems involved in long COVID symptomatology.

This study also validates dysfunctional TRPM3 as a consistent biomarker of long COVID as it is the third cohort of long COVID participants exhibiting impaired TRPM3 ion channel function after modulation with PregS and ononetin (Sasso et al., 2022; Sasso et al., 2024). In all three cohorts of individuals with long COVID (not on treatment with LDN), TRPM3 consistently showed a significant loss of function, characterized by reduced current amplitudes in response to PregS and an increase in the number of NK cells resistant to ononetin compared to HC groups, as illustrated in Figures 1D–F, 2A-C.

In recent years, growing evidence from studies and guidelines has provided recommendations to manage acute COVID-19 infection both in hospitalized and non-hospitalized patients. However, there are very few options for the treatment of those individuals who developed long COVID (The Lancet Infectious Diseases, 2023; Bonilla et al., 2023). This investigation provides novel evidence supporting the potential therapeutic benefits of LDN for individuals living with long COVID. Our findings are in agreement with those of a previous study that investigated the *in vitro* effects of NTX on NK cells from long COVID patients (Sasso et al., 2024). In that study, following a 24-h incubation with 200 μM NTX, TRPM3 function in NK cells from long COVID patients was restored to levels comparable to those of the HC group during stimulation with agonist and antagonist drugs, as assessed using whole-cell patch-clamp. Hence, the previous study demonstrated that NTX reversed TRPM3 dysfunction and restored intracellular Ca^2+^ concentration, thereby facilitating homeostatic cellular processes (Sasso et al., 2024). In both the previous study (following NTX treatment in culture) and the current study (long COVID patients receiving LDN), TRPM3 currents in NK cells from long COVID patients were restored to levels similar to those observed in HC individuals.

NTX has been shown to exert a wide range of effects on cells. At doses below 5 mg, NTX plays a significant function as a glial modulator within the central nervous system, partially suppressing opioid receptor signaling and exerting neuroprotective effects by inhibiting microglial activation (Trofimovitch and Baumrucker, 2019; Kucic et al., 2021). LDN blocks Toll-like receptor 4 (TLR4), which is highly expressed in microglial cells. By modulating TLR4 signaling, LDN helps attenuate the downstream production of pro-inflammatory cytokines, thereby reducing neuroinflammation (Kucic et al., 2021; Marcus et al., 2024; Isman et al., 2024). Parkitny et al. associated LDN treatment with a reduction in the plasmatic concentrations of several inflammatory cytokines in women with fibromyalgia. These included interleukin (IL)-1β, IL-1Ra, IL-2, IL-4, IL-5, IL-6, IL-10, IL-12p40, IL-12p70, IL-15, IL-17A, IL-27, interferon-α, transforming growth factor (TGF)-α, TGF-β, tumor necrosis factor-α, and granulocyte-colony stimulating factor (Parkitny and Younger, 2017). Importantly, the increase in endogenous opioid production modulates the immune system, while the inhibition of the opioid growth factor receptor by LDN causes a feedback loop response that elevates endogenous opioid signaling. This response is associated with the development and functioning of tissues and organs (Marcus et al., 2024; Isman et al., 2024). The increase in endogenous opioids is associated with modulating the immune system through an inhibitory role in B and T cell proliferation (Patten et al., 2018).

Notably, as a μ-opioid receptor antagonist, NTX counteracts the inhibitory function of opioid receptors on TRPM3 ion channels, thereby restoring TRPM3 function and reestablishing Ca^2+^ influx (Eaton-Fitch et al., 2022; Cabanas et al., 2019b; Sasso et al., 2024). Typically, upon activation, opioid receptors bind to heterotrimeric Gi/o proteins, which then dissociate into Gαi/o and Gβγ subunits, subsequently modulating diverse intracellular signaling pathways (Machelska and Celik, 2018). Gβγ protein has the ability to activate various K^+^ channels, but it blocks TRPM3 ion channels and voltage-gated Ca^2+^ channels (Heinke et al., 2011; Marker et al., 2005; Dembla et al., 2017). Interestingly, the effects of opioids are not confined to the brain, as opioid receptors are broadly distributed in tissues and organ systems, including immune cells. Therefore, opioids can exert both immunomodulatory and immunosuppressive effects (Ninkovic and Roy, 2013).

Although our research primarily focuses on the TRPM3 ion channel to elucidate its specific role in the pathomechanism of long COVID and its potential as a therapeutic target, other TRP ion channels may also contribute to this complex illness. While there is no consensus on pathophysiology mechanisms underlying long COVID, a number of hypotheses have been proposed. These include immune dysregulation after viral infection, leading to widespread inflammation across multiple organs, autoimmunity, microthrombosis caused by hypercoagulation, reactivation of latent pathogens, persistent virus reservoirs, and mitochondrial dysfunction (Al-Aly et al., 2024; Altmann et al., 2023; Dietz and Brondstater, 2024; Hurt et al., 2024; Sherif et al., 2023). Interestingly, TRP ion channels are significant contributors to the regulation of inflammation and immune functions by modulating intracellular Ca^2+^ concentration, which are vital for immune cell responses and the maintenance of inflammation homeostasis in health and disease (Clement et al., 2020; Parenti et al., 2016; Silverman et al., 2020). TRPM2 and TRPM7 are TRP channels involved in the modulation of oxidative stress and immune function. Disruptions in TRPM2 and TRPM7 have been linked to the pathomechanism of ME/CFS (Yamamoto et al., 2008; Du et al., 2021; Balinas et al., 2019; Du Preez et al., 2023). Elevated TRPV1 mRNA has been observed in ME/CFS patients following exercise, reflecting its role in pain perception and heat sensitivity (White et al., 2012). Building on these findings from ME/CFS research, future investigations into these TRP channels in long COVID may provide further insights into the overlapping pathomechanisms between these diseases, as well as uncover potential new therapeutic interventions.

TRP channels are activated in multiple ways, including environmental or mechanical stimuli, natural products, and endogenous agents and messengers released during tissue injury and inflammation (Parenti et al., 2016; Liu and Montell, 2015; Zheng, 2013; Kaneko and Szallasi, 2014; Khalil et al., 2018). In addition, TRP channels are widely expressed in various tissues affected by SARS-CoV-2 infection and have been implicated in a range of symptoms associated with COVID-19, such as pain, fever, inflammation, loss of smell and taste, and multiple system symptoms (gastrointestinal, cardiovascular, respiratory, and neurological) (Jaffal and Abbas, 2021), which are also often observed in individuals with long COVID (Davis et al., 2021; Lopez-Leon et al., 2021; Carfi et al., 2020). Given their involvement in multiple pathogenic processes, TRP ion channels have emerged as promising targets for pharmacotherapeutic interventions (Clement et al., 2020; Hasan and Zhang, 2018).

Even though the primary outcome of this research was to assess TRPM3 ion channel function, we also analyzed data on QoL and disability from participants. Significant differences were observed across all WHODAS domains and six out of eight SF-36 domains (except emotional role and emotional well-being). The HC group exhibited higher scores on the SF-36, indicating better QoL and lower scores on the WHODAS, suggesting less disability compared with both long COVID groups (with and without LDN). Although the long COVID group receiving LDN showed higher SF-36 scores in seven of eight domains (except Emotional Role) and lower WHODAS scores across all domains compared to the long COVID group, a significant difference was found only in the mobility domain (p = 0.029) between the two long COVID groups. Interestingly, there were no significant differences between the HC and the long COVID group receiving LDN in SF-36 scores in four of eight domains (general health, emotional role, emotional well-being, and vitality) and in WHODAS scores from three of seven domains (self-care, interpersonal relationships, and work activities). Our results provide further evidence of LDN’s safety, as all scores from the SF-36 and WHODAS showed a tendency for improvement, and this study did not indicate any harmful effects of LDN treatment for long COVID patients.

Growing evidence has shown significant improvement in symptoms, QoL, and disability in long COVID patients receiving LDN, supporting its effectiveness as a treatment for long COVID. For instance, O’Kelly et al. reported that long COVID patients experienced improvements in six of seven parameters assessed, with a satisfactory safety range of 94.7% (O'Kelly et al., 2022). A cross-sectional follow-up study conducted at a long COVID clinic found that 58% of patients treated with LDN reported eased symptoms (Hurt et al., 2024). The findings by Bonilla et al. linked LDN treatment to a reduction in the number of symptoms experienced by long COVID patients, relief of clinical symptoms, and improved function performance (Bonilla et al., 2023). Another investigation evaluated symptoms and QoL in long COVID patients treated with LDN and nicotinamide adenine dinucleotide (NAD^+^) supplementation, suggesting a significant improvement in fatigue symptoms and QoL after 12 weeks compared to before LDN and NAD^+^ treatment (Isman et al., 2024). It is evident that there is a shortage of well-designed clinical trials or studies focused on understanding the mechanism of LDN in long COVID. This novel finding supports the use of off-label LDN to treat individuals with long COVID. However, future clinical trials should evaluate symptom presentation, QoL, and disability in a larger cohort of long COVID patients. Although LDN is a safe medication associated with improvements in wellbeing and mitigation of long COVID symptoms, double-blind, randomized clinical trials are essential to examine the benefits of LDN treatment (Patten et al., 2018; O'Kelly et al., 2022; Isman et al., 2024).

The consequences of long COVID are not restricted to the affected individuals and their families but have a broad impact on health and economic systems (Al-Aly et al., 2024; Costantino et al., 2024). Therefore, identifying effective therapies to improve long COVID management is crucial to mitigate this public health crisis. The novel findings from this investigation indicate a significant restoration of TRPM3 ion channel function in NK cells from long COVID patients treated with LDN. Our current data aligns with previous research and provides evidence of impaired TRPM3 as a consistent biomarker in NK cells from long COVID patients, suggesting the involvement of channelopathy in the pathophysiology of this condition. These results also support LDN as a safe therapeutic intervention to restore TRPM3-dependent Ca^2+^ influx and facilitate cellular functions in long COVID patients. However, further investigation through well-designed clinical trials is needed to confirm the effects of LDN on improving symptoms, disability, and QoL in long COVID.

## Data Availability

The datasets generated and analysed for this study are not publicly available due to confidentiality agreements, but can be made available upon reasonable request. Requests to access the datasets should be directed to ncned@griffith.edu.au.

## Abbreviations

BMI, body mass index; Ca^2+^, calcium; EDTA, ethylenediaminetetraacetic acid; FBC, Full Blood Count; GPCR, guanine nucleotide-binding protein-coupled receptors; HC, healthy controls; IL, interleukin; LDN, low‐dose naltrexone; ME/CFS, myalgic encephalomyelitis/chronic fatigue syndrome; NAD+, nicotinamide adenine dinucleotide; NCNED, National Centre for Neuroimmunology and Emerging Diseases; NK, natural killer; NTX, naltrexone; PBMC, peripheral blood mononuclear cells; PregS, pregnenolone sulfate; QoL, quality of life; SARS-CoV-2, respiratory syndrome coronavirus 2; SD, standard derivation; SF-36, 36-item Short-Form Health Survey; SPSS, Statistical Package for the Social Sciences; TGF, transforming growth factor; TLR4, Toll-like receptor 4; TRPM3, Transient Receptor Potential Melastatin 3; WHO, World Health Organization; WHODAS, World Health Organization Disability Assessment Schedule.
