# Supplementary figures and images for: Low‐Dose naltrexone restored TRPM3 ion channel function in natural killer cells from long COVID patients
Source: Front Mol Biosci. 2025 May 19;12:1582967. doi: 10.3389/fmolb.2025.1582967 (PMC12127304; doi:10.3389/fmolb.2025.1582967)

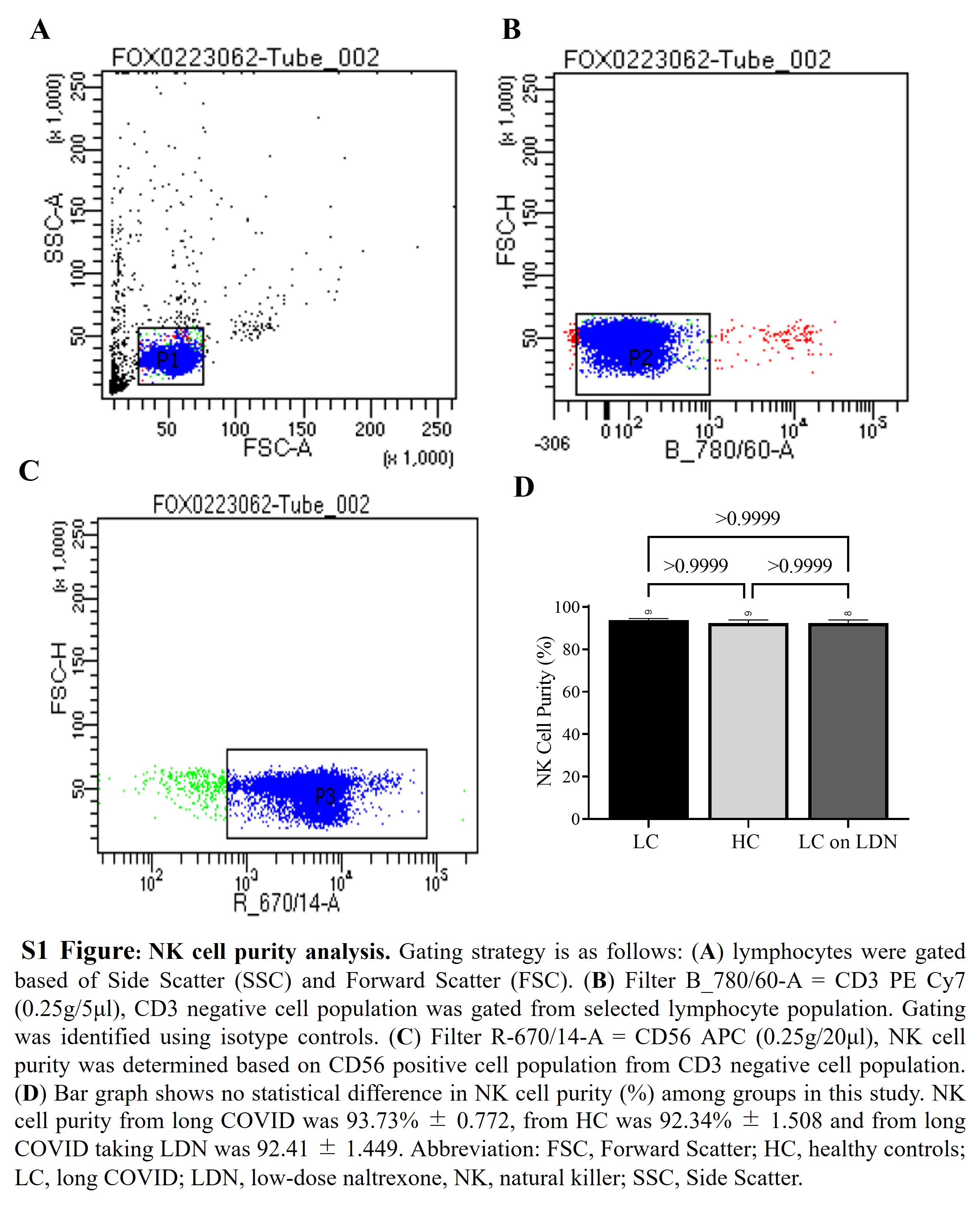

Supplement: Supplementary file 1 [file Image1.tiff]
